# A New Class of PSMA-617-Based Hybrid Molecules for Preoperative Imaging and Intraoperative Fluorescence Navigation of Prostate Cancer

**DOI:** 10.3390/ph15030267

**Published:** 2022-02-22

**Authors:** Ann-Christin Eder, Jessica Matthias, Martin Schäfer, Jana Schmidt, Nils Steinacker, Ulrike Bauder-Wüst, Lisa-Charlotte Domogalla, Mareike Roscher, Uwe Haberkorn, Matthias Eder, Klaus Kopka

**Affiliations:** 1 Department of Radiopharmaceutical Chemistry, German Cancer Research Center (DKFZ), 69120 Heidelberg, Germany; martin.schaefer@dkfz-heidelberg.de (M.S.); janageberlein@gmail.com (J.S.); u.bauder-wuest@dkfz-heidelberg.de (U.B.-W.); mareike.roscher@dkfz-heidelberg.de (M.R.); k.kopka@hzdr.de (K.K.); 2 Department of Nuclear Medicine, University Medical Center Freiburg, Faculty of Medicine, University of Freiburg, 79106 Freiburg, Germany; nils.steinacker@uniklinik-freiburg.de (N.S.); lisa-charlotte.domogalla@uniklinik-freiburg.de (L.-C.D.); matthias.eder@uniklinik-freiburg.de (M.E.); 3 Department of Radiopharmaceutical Development, German Cancer Consortium (DKTK), Partner Site Freiburg, Freiburg, Germany and German Cancer Research Center, 69120 Heidelberg, Germany; 4 Department of Optical Nanoscopy, Max Planck Institute for Medical Research, 69120 Heidelberg, Germany; jmatthi@mr.mpg.de; 5 Department of Nuclear Medicine, Heidelberg University Hospital, 69120 Heidelberg, Germany; uwe.haberkorn@med.uni-heidelberg.de; 6 Clinical Cooperation Unit Nuclear Medicine, German Cancer Research Center (DKFZ), 69120 Heidelberg, Germany; 7 Helmholtz-Zentrum Dresden-Rossendorf (HZDR), Institute of Radiopharmaceutical Cancer Research, 01328 Dresden, Germany; 8 German Cancer Consortium (DKTK), Partner Site Dresden, 01328 Dresden, Germany; 9 School of Science, Faculty of Chemistry and Food Chemistry, Technical University Dresden, 01069 Dresden, Germany

**Keywords:** PSMA, hybrid molecules, prostate cancer, guided surgery, theranostics

## Abstract

The development of PSMA-targeting low-molecular-weight hybrid molecules aims at advancing preoperative imaging and accurate intraoperative fluorescence guidance for improved diagnosis and therapy of prostate cancer. In hybrid probe design, the major challenge is the introduction of a bulky dye to peptidomimetic core structures without affecting tumor-targeting properties and pharmacokinetic profiles. This study developed a novel class of PSMA-targeting hybrid molecules based on the clinically established theranostic agent PSMA-617. The fluorescent dye-bearing candidates of the strategically designed molecule library were evaluated in in vitro assays based on their PSMA-binding affinity and internalization properties to identify the most favorable hybrid molecule composition for the installation of a bulky dye. The library’s best candidate was realized with IRDye800CW providing the lead compound. Glu-urea-Lys-2-Nal-Chx-Lys(IRDye800CW)-DOTA (PSMA-927) was investigated in an in vivo proof-of-concept study, with compelling performance in organ distribution studies, PET/MRI and optical imaging, and with a strong PSMA-specific tumor uptake comparable to that of PSMA-617. This study provides valuable insights about the design of PSMA-targeting low-molecular-weight hybrid molecules, which enable further advances in the field of peptidomimetic hybrid molecule development.

## 1. Introduction

The development of the theranostic agent PSMA-617, a prostate-specific membrane antigen (PSMA) inhibitor with highly specific tumor enhancement and superior pharmacokinetics, has remarkably advanced diagnostics and therapy of prostate cancer [1]. PSMA-617 can be labeled with positron-emitters (e.g., ^68^Ga) for imaging or with radionuclides suitable for therapy (e.g., ^177^Lu, ^225^Ac) of recurrent prostate carcinoma [2,3]. In particular, the rapid renal elimination profile of PSMA-617 renders the molecule advantageous for therapeutic approaches, as its enrichment in non-malignant tissue, and therefore the associated radiation exposure, is reduced to a minimum.

Efficacy, safety, and quality of life after [^177^Lu]Lu-PSMA-617 therapy have been evaluated in a phase II clinical trial [4]. At twelve weeks after the first dose, half of the patients experienced a prostate-specific antigen (PSA) decline greater than 50%, whereas for 27% of the patients, the PSA decline was even greater than 80%. The currently most advanced clinical trial is a recently completed phase III study (VISION) comprising 831 patients. Published data suggest that [^177^Lu]Lu-PSMA-617 significantly improves overall survival while the treatment regimen is very well-tolerated [5]. Hence, the FDA approval of radioligand therapy with [^177^Lu]Lu-PSMA-617 is expected shortly. Due to its outstanding clinical performance and its favorable pharmacokinetic profile, PSMA-617 represents an ideal core structure for hybrid ligand development in the field of prostate cancer [4,5].

In the case of localized and locally advanced prostate cancer, radical prostatectomy with lymph node dissection and lymphadenectomy is an established curative strategy [6]. Preoperatively, the identification of primary tumors and metastases can be performed precisely with PSMA-targeting diagnostic radiopharmaceuticals by PET/CT imaging. However, the accurate intraoperative localization and delineation of tumor margins or lymph node metastases is still a major challenge in oncological surgery in general. For prostate carcinoma, the established workaround is a template-based removal of nonpathological lymph nodes with the drawback of increased morbidity. Additionally, there is an enhanced risk of residual tumorous structures [7,8]. Targeted hybrid molecules comprising both radio- and fluorescent label promise to overcome these limitations by supporting pre- as well as intraoperative navigation. The radioactive label allows for preoperative cancer staging and surgery planning by noninvasive imaging (e.g., PET/CT), and the fluorescent label subsequently provides visual intraoperative guidance.

For clinical applications, low-molecular-weight peptidomimetics provide the ideal molecular format. Due to fast clearance from nontarget organs and high tumor penetration and retention, such molecules yield clear imaging contrasts at early time points after injection, simplifying clinical protocols. Recently, the first PSMA-targeting peptidomimetic low-molecular-weight hybrid agents have been developed [9,10,11,12]. With the research field still in its infancy, the first clinical application of the PSMA-11-derived peptidomimetic PSMA-targeting molecule PSMA-914 already highlights the great future potential of targeted hybrid agents in the surgical treatment of prostate cancer patients and emphasizes the urgent need for further preclinical development and clinical studies [13].

From a preclinical perspective, the development of novel hybrid molecules based on preceding generations allows to further improve the molecules’ properties (tumor targeting, pharmacokinetics) by benefitting from previously gained knowledge, in particular about structure–activity relationships. Here, the major challenge in hybrid probe design is to preserve a fast pharmacokinetic profile (fast distribution and clearance rate) as well as high and specific tumor targeting properties when introducing a bulky dye to the comparatively small low-molecular-weight peptidomimetic.

This preclinical proof-of-concept study aimed at identifying favorable molecule compositions for the installation of bulky dyes to further fuel the field of hybrid probe development. We present a novel class of PSMA-targeting hybrid molecules based on the clinically established theranostic agent PSMA-617 [1]. Introducing the dual-label to the core structure via a lysine branch resulted in a strategically designed molecule library.

In the first step, the ideal hybrid molecule composition (dye position in the molecule, additional introduction of glutamic acid spacer moieties) was identified via its PSMA-binding properties and internalization profiles. In the second step, the clinically relevant IRDye800CW was introduced to the library’s best candidate to form the lead compound of this study. IRDye800CW was chosen over its clinically approved alternative indocyanine green, as the latter is prone to human serum albumin binding, which tremendously slows down pharmacokinetics [14,15]. In the third step, the lead compound’s tumor uptake and pharmacokinetic properties were determined.

## 2. Results

### 2.1. Synthesis, Radiolabeling, and Spectral Properties

**Figure 1:**
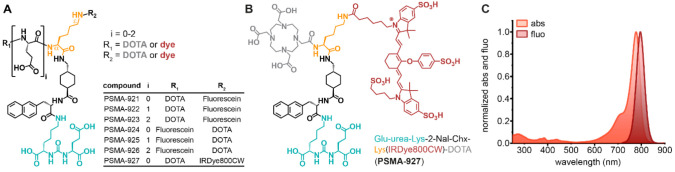
Chemical structures and spectral properties of PSMA-617-based hybrid molecules. (**A**) Generic structure with a lysine branch (orange), glutamic acid linker moieties (i = 0–2), and fluorescent dye (red) and DOTA (gray) conjugated as R_1_ or R_2_. (**B**) The lead candidate of the molecule library consists of the PSMA-binding motif “Glu-urea-Lys” (cyan), the radiometal chelator “DOTA” (gray), and the fluorescence moiety “IRDye800CW” (red) conjugated via a “2-Nal-Chx-Lys” linker moiety (black and orange). (**C**) Absorption and emission spectra of PSMA-927 peaking at 778 and 796 nm, respectively.

For the synthesis of a PSMA-targeting hybrid molecule library based on PSMA-617 [1], a lysine branch was introduced to the core structure to modularly install a fluorescent dye and radionuclide chelator. The dye was conjugated to the lysine either via the amino group in the α-position or to the amino group in the ԑ-position of the side chain. The chelator was positioned accordingly at the free amino group. Additionally, the influence of glutamic acid (E) as a linker was investigated through repetitive insertion (i = 0–2) (Figure 1A). To identify the most favorable hybrid framework via in vitro characterization, the library was realized with fluorescein as dye and DOTA as chelator. Finally, fluorescein was replaced in the best-performing core hybrid structure by the clinically relevant NIR-dye IRDye800CW, yielding the lead candidate PSMA-927 (Figure 1B).

Seven compounds were synthesized via solid-phase and classical organic synthesis strategies. Fluorescein isothiocyanate (FITC) and IRDye800CW were conjugated to the peptidomimetic precursor molecules, resulting in final products with purities >98%. The detailed synthetic strategies and analytical data of the final compounds are provided in Supplementary Materials (Supplemental Scheme S1, Scheme S2, Scheme S3 and Supplemental Table S1). Determined as logD at pH 7.4 in n-octanol/PBS, the compounds’ lipophilicities were comparable to the one of the reference compound [^68^Ga]Ga-PSMA-617 (log*D*_pH 7.4_: –2.00) (Supplemental Table S1) [1]. Radiochemical yields obtained were >99% after radiolabeling with ^68^Ga (Supplemental Figure S1, Supplementary Materials). For PSMA-927, spectral characterization revealed an absorption maximum of 778 nm and an emission maximum of 796 nm, very similar to those of the free dye (abs 775 nm, em 796 nm) (Figure 1C) [16].

### 2.2. In Vitro Evaluation: PSMA-Specific Binding and Internalization Profile

For all tested molecule compositions, introduction of fluorescein to PSMA-617 via a lysine branch always kept the PSMA-binding affinity in the low nanomolar range comparable to the one of PSMA-617 (K_i_ = 2.34 ± 2.94 nmol/L) (Table 1) [1]. Thus, the PSMA-specific internalization profiles of the fluorescein-conjugated hybrid molecules were determined to identify the most favorable position in the molecular framework for dye installation. Interestingly, introduction of the dye at the α-position to the carboxy group of the lysine led to a substantial decrease in specific cell surface binding and internalization. However, the observed effect was reversible upon insertion of a glutamic acid linker between the lysine and the dye, and the increase in cell surface binding and internalization even correlated with the number of inserted glutamic acid repetitions (Table 1).

In contrast to the dye installation at the α-position, fluorescein conjugation to the amino group in the ԑ-position of the lysine side chain was well-tolerated and retained high cell surface binding and internalization properties comparable to those of the PSMA-617 internalization profile ([^68^Ga]Ga-PSMA-617 [18]: cell surface binding of 11.40 ± 6.60 %ID/10^5^ LNCaP cells, specific internalization of 16.17 ± 3.66 %ID/10^5^ LNCaP cells). The addition of glutamic acid repetitions between the amino group in the ԑ-position and fluorescein did not significantly affect the internalization profile. Consequently, to provide the most compact lead compound, IRDye800CW was conjugated to that very position without an additional amino acid linker.

The binding affinity of PSMA-927 was in the low nanomolar range, but slightly decreased compared to that of its fluorescein counterpart (Table 1). However, [^68^Ga]Ga-PSMA-927 featured the highest specific cell surface binding (25.51 ± 9.73 %ID/10^5^ LNCaP cells) and specific internalization rate (27.64 ± 12.80 %ID/10^5^ LNCaP cells) of all library candidates, similar to those of the profile of [^68^Ga]Ga-PSMA-617 [18] (Table 1).

**Figure 2:**
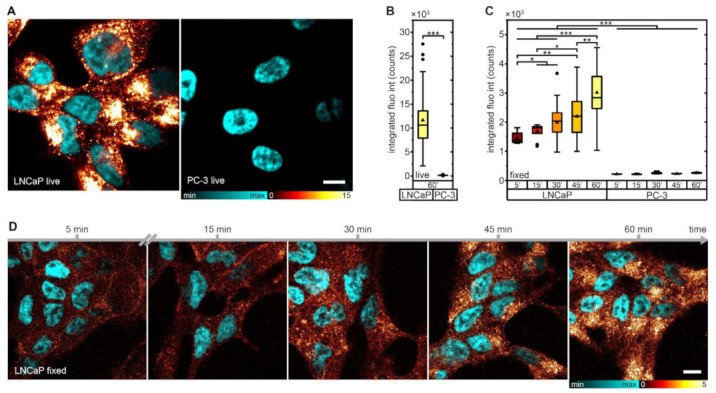
In vitro fluorescence imaging of PSMA-927. (**A**) Confocal images of living LNCaP and PC-3 cells after incubation with 50 nmol/L compound for 60 min; scale bar, 10 µm. (**B**) Integrated fluorescence intensity of LNCaP and PC-3 cells after incubation with 50 nmol/L compound for 60 min without fixation and (**C**) for 5/15/30/45/60 min with PFA fixation. Note that imaging parameters differed between live and fixed cell imaging and thus the integrated fluorescence intensity could not be directly compared (LNCaP: live 60 min n = 54, fixed 5 min *n* = 10, fixed 15 min *n* = 10, fixed 30 min *n* = 21, fixed 45 min *n* = 22, fixed 60 min *n* = 19; PC-3: live 60 min *n* = 32, fixed 5 min *n* = 5, fixed 15 min *n* = 5, fixed 30 min *n* = 5, fixed 45 min *n* = 5, fixed 60 min *n* = 11). Significance: * *p* < 0.05; ** *p* < 0.01; *** *p* < 0.001. (**D**) Confocal images of fixed LNCaP cells after incubation with 50 nmol/L compound for 5/15/30/45/60 min; scale bar, 10 µm.

Fluorescence microscopy experiments confirmed the in vitro data that were based on the compound’s radioactivity. In live cell confocal imaging, PSMA-927showed PSMA-specific cell binding and fast internalization. While imaging contrasts peaked for PSMA-positive LNCaP cells at 1 h of internalization time, no significant fluorescence signal increase was detected with PSMA-negative PC-3 cells (Figure 2A,B). To display the increasing internalized fraction of the compound over internalization time, fixed-cell confocal data were collected (Figure 2C,D). Due to the sensitivity of the compound’s fluorescence properties to fixation, the deposited dosage of 775 nm excitation light in the sample had to be decreased by a factor of three in comparison to that in the live-cell imaging experiments. The ratio of the integrated fluorescence intensities for live and fixed data after 60 min of internalization clearly reflected the adapted imaging parameters (integrated fluorescence intensity live 11,618 ± 5412 counts, integrated fluorescence intensity fixed 3021 ± 863 counts, ratio = 3.8 ± 2.1).

**Figure 3:**
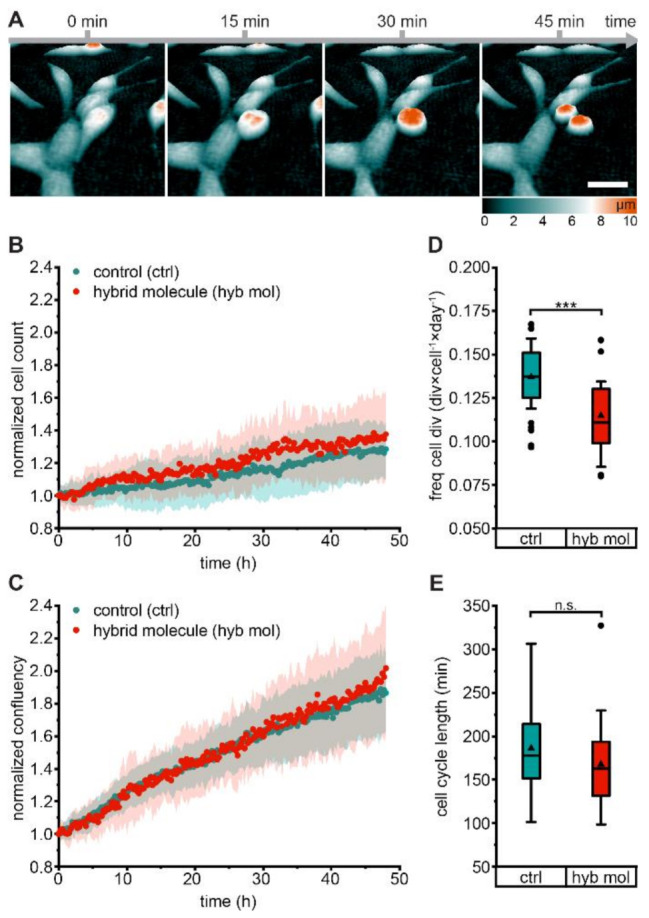
Holographic time-lapse imaging of LNCaP cell proliferation under compound exposure. LNCaP cell proliferation was followed for a total period of 48 h in the absence of (control, cyan, experiments *n* = 38, tracked cells *n* = 33,031, divisions *n* = 9157, Supplemental Movie S1) and in the constant presence of 100 nmol/L PSMA-927 (hybrid molecule, red, experiments *n* = 25, tracked cells *n* = 35,804, divisions *n* = 8421) via holographic time-lapse cytometry. (**A**) Holographic image sequences of a cell division in the presence of compound. Dividing cells rounded up and could be distinguished from nondividing cells by height (color-coded); scale bar, 50 μm. A corresponding time-lapse movie (Supplemental Movie S2) is supplied. (**B**) Normalized cell count (average *p*-value 0.235 ± 0.227) and (**C**) normalized confluency (average *p*-value 0.546 ± 0.251) of untreated and treated LNCaP cells. (**D**) Frequency of cell division and (**E**) cell cycle length of untreated and treated LNCaP cells. Significance: *** *p* < 0.001; ns, no significant differences.

To assess potential cytotoxic effects of PSMA-927, LNCaP cell proliferation was monitored by holographic time-lapse imaging during constant compound exposure for 48 h (Figure 3A, Supplemental Movie S1 and Movie S2). Analysis of cell count (Figure 3B), confluency (Figure 3C), frequency of cell division (Figure 3D), and cell cycle length (Figure 3E) did not reveal any evidence of cytotoxicity. We attributed the slightly reduced cell division frequency to the biological variation that was expected for this in vitro assay, as it was not reflected in a decreased cell count. Nevertheless, the data are in good agreement with previous cytotoxicity studies of STED-compatible hybrid PSMA-inhibitors [19].

Taken together, these favorable in vitro characteristics qualified PSMA-927 as the lead compound for further evaluation in a subsequent preclinical proof-of-concept study.

### 2.3. In Vivo Proof-of-Concept Study: PSMA-Mediated Specific Uptake in Xenograft Tumors

**Figure 4:**
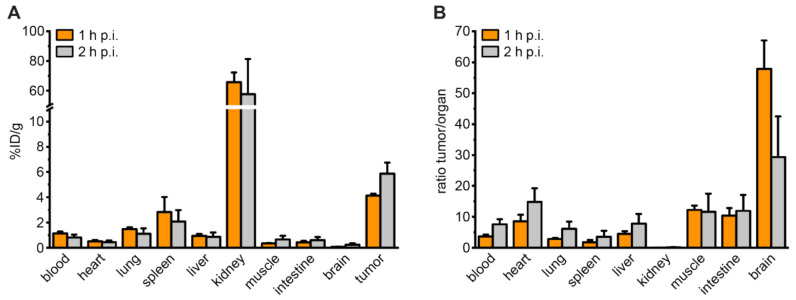
Organ distribution of ^68^Ga-labeled PSMA-927. (**A**) Organ distribution of 60 pmol ^68^Ga-labeled compound at 1 and 2 h p.i. and (**B**) corresponding LNCaP-tumor-to-organ ratios of 60 pmol ^68^Ga-labeled compound at 1 and 2 h p.i. in LNCaP-tumor bearing BALB/c nu/nu mice. Data are expressed as mean % ID/g tissue ± SD (*n* = 3).

In biodistribution studies, ^68^Ga-labeled PSMA-927 showed high tumor accumulation of 4.13 ± 0.15% ID/g at 1 h p.i., which was reduced but statistically still comparable to the tumor uptake of [^68^Ga]Ga-PSMA-617 (8.47 ± 4.09% ID/g, *p* > 0.5) (Figure 4A, Supplemental Table S2 and Table S3, Supplementary Materials) [1]. The biodistribution profile was constant up to 2 h p.i. with a tumor accumulation of 5.85 ± 0.91% ID/g at 2 h p.i. (Figure 4A) leading to high tumor-to-organ ratios (Figure 4B, Supplemental Table S2 and Table S3, Supplementary Materials).

**Figure 5:**
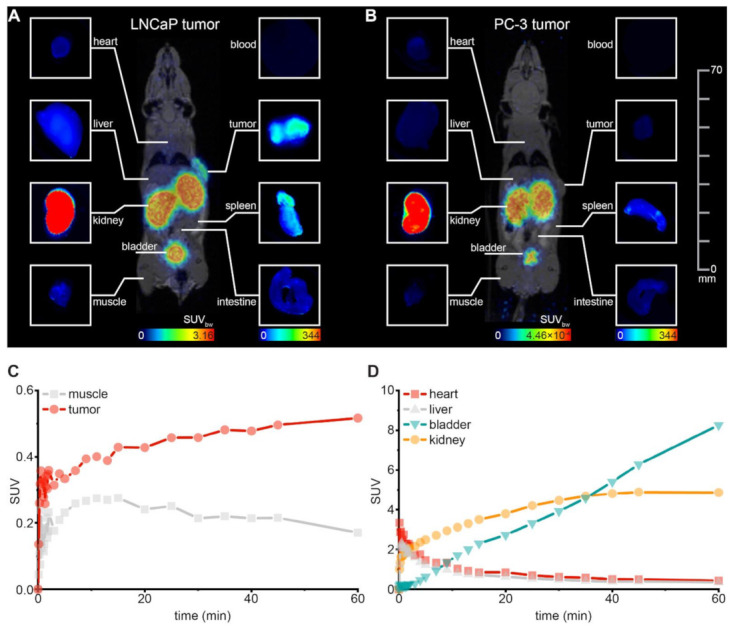
Small-animal proof-of-concept study. Whole-body maximum intensity projections of 0.5 nmol ^68^Ga-labeled PSMA-927 in (**A**) LNCaP- and (**B**) PC-3-tumor-bearing BALB/c nu/nu mice (right flank) 120 min p.i. obtained from small-animal PET/MR imaging (each n = 1) and corresponding fluorescence images of organs dissected after PET/MRI (acquired with the Odyssey CLx system; excitation wavelength 800 nm). For the PSMA-positive LNCaP-tumor, corresponding time activity curves are shown (**C**) for tumor and muscle and (**D**) for background organs. SUV = standardized uptake value.

The strong and specific tumor uptake was confirmed by a small animal PET/MRI study with [^68^Ga]Ga-PSMA-927 in a PSMA-positive LNCaP xenograft mouse model (Figure 5A). The compound’s high PSMA-specificity was additionally demonstrated by the lack of measurable uptake in PSMA-negative PC-3 tumor xenografts (Figure 5B). The pharmacokinetic profile was characterized by rapid clearance from non-malignant tissue (heart, liver, muscle) via the renal pathway (Figure 5C,D).

After PET/MRI, fluorescence imaging of the individual organs visualized the high tumor uptake of [^68^Ga]Ga-PSMA-927 with strong tumor-to-background contrasts confirming the results that were acquired beforehand based on radioactivity (Figure 5A,B).

## 3. Discussion

A new class of PSMA-targeting hybrid molecules derived from the clinically established theranostic agent PSMA-617 was introduced for imaging and therapy of prostate cancer. Due to its highly specific tumor enrichment and favorable pharmacokinetic profile, PSMA-617 was selected as a well-suited core structure for the development of dual-labeled PSMA-inhibitors [1,4,5]. The hybrid modality of radionuclide chelator and fluorescent dye was installed by branching the molecule with the amino acid lysine. The amino groups in the α-position and in the ԑ-position of the side chain were selected for conjugation of dye or chelator. The molecule library was extended by repetitively inserting a glutamic acid (E) linker (i = 0–2) between dye and core, and chelator and core. Initially, fluorescein was installed as a fluorescent dye to identify the best-performing core structure of the novel series of hybrid molecules in an in vitro evaluation.

PSMA binding was affected neither by the branching nor by fluorescein incorporation, with affinities residing in the low nanomolar range for all library compounds. In contrast, the arrangement of dye and chelator had a significant effect on the internalization profiles. With DOTA positioned at the amino group in the ԑ-position of the lysine side chain, compound internalization was impaired. Remarkably, this effect could be reversed by introducing a glutamic acid linker moiety. As DOTA in the ԑ-position of the lysine side chain still allowed for a high PSMA binding affinity, we concluded that this specific molecule composition indirectly affected the process of clathrin-based endocytosis, which is known to mediate PSMA-inhibitor uptake [19,20]. As the efficiency of PSMA internalization is suggested to depend on the extent of PSMA conformational changes upon PSMA-inhibitor binding, we assumed that DOTA in the ԑ-position of the lysine side chain significantly altered these conformational changes [20,21]. An influence of charges could be excluded, as all compounds carrying the dye with and without a glutamic acid linker moiety in the ԑ-position of the side lysine chain internalized comparably to the reference PSMA-617.

Based on the in vitro characterization of this hybrid molecule library, Glu-urea-Lys-2-Nal-Chx-Lys(dye)-DOTA was selected as the most favorable core structure for the conjugation of the clinically relevant NIR-dye IRDye800CW due to its high affinity to PSMA, specific internalization, and the lowest molecular weight amongst all library compounds. The lead compound PSMA-927 showed an increased and specific internalization rate, but a slight yet insignificant decrease in binding affinity compared to that of all library candidates.

These results were supported by confocal imaging experiments. Both live- and fixed-cell data confirmed PSMA-specific internalization and an increasing compound accumulation over time. Strikingly, PSMA-927 performed significantly better in terms of photostability under live-cell imaging conditions, further qualifying it for intraoperative navigation. Additionally, cytotoxicity of the compound could be excluded by in vitro holographic time-lapse imaging.

The advantageous internalization profile resulted in a high PSMA-specific tumor accumulation with high tumor-to-organ ratios stable up to 2 h p.i. and comparable to the ones of [^68^Ga]Ga-PSMA-617 [1]. Notably, the low uptake of PSMA-617 in the spleen was preserved even after dye conjugation. Only the renal clearance of the novel hybrid molecule appeared to be slightly delayed compared to PSMA-617 clearance when referring to time-activity curves up to 1 h p.i. [1]. This retarding effect might be attributed to the addition of the bulky NIR-dye. As the molecule’s accumulation in organs close to the prostate can impede cancer detection, subsequent studies accelerating the molecule’s renal clearance are of interest.

Comparing our lead compound to former generations of PSMA-targeting hybrid molecules, we concluded that a PSMA-11-based core structure tolerated dye conjugation better than its PSMA-617-based counterpart, as the HBED compounds [^68^Ga]Ga-Glu-urea-Lys-HBED-CC-IRDye800CW and [^68^Ga]Ga-Glu-urea-Lys-(HE)_3_-HBED-CC-IRDye800CW exhibited significantly higher tumor enhancement after dye conjugation [11,12]. Nevertheless, the novel hybrid molecule PSMA-927 revealed great tumor-to-background ratios in PET/MRI and optical imaging at early time points, which is crucial for sufficient contrast in a surgical setting. In particular, high tumor-to-blood and tumor-to-muscle ratios are critical for precise delineation between tumor and non-malignant tissue.

Taken together, we present PSMA-927 as the lead compound of the first PSMA-617-based hybrid molecule generation, which supports PSMA-617 as a suitable core structure for future hybrid molecule development. With its high tumor uptake, fast clearance from nontarget organs, and nontoxic profile, the novel PSMA-targeting hybrid molecule qualifies for a clinical translation and strongly encourages realization and extended preclinical investigation of related PSMA-inhibitor designs.

## 4. Materials and Methods

### 4.1. Chemical Synthesis, Radiolabeling, and Determination of Lipophilicity

A series of linker modifications was introduced to PSMA-617, providing suitable core structures for fluorescent dye conjugation to create PSMA-617-derived hybrid molecules. The synthesis was performed according to previously published protocols [11,17,18,22]. Details on synthesis and analytical data of the compounds are provided in the Supplementary Materials (Compound Synthesis and Analytical Characterization). For labeling with ^68^Ga, the precursor peptide (1 nmol in 2-[4-(2-hydroxyethyl)piperazin-1-yl]ethanesulfonic acid (HEPES, Sigma-Aldrich, Darmstadt, Germany) buffer (580 mg/mL) with 5 mg ascorbic acid, Sigma-Aldrich, Darmstadt, Germany, 90 µL) was added to 40 µL of ^68^Ga^3+^ eluate (~40–60 MBq). Using 30% NaOH (Sigma-Aldrich, Darmstadt, Germany) and 10% NaOH (Sigma-Aldrich, Darmstadt, Germany), the pH was adjusted to 3.8. The reaction mixture was incubated at 98 °C for 5–10 min. The radiochemical yield was analyzed by reversed-phase high-performance liquid chromatography (RP-HPLC) or reversed-phase thin-layer chromatography (RP-TLC, 60 RP-18 F254S, Sigma-Aldrich, Darmstadt, Germany) with 0.5 M ammonium acetate/methanol (1:1) as the mobile phase. The lipophilicity of the ^68^Ga-labeled compounds was determined using the 2-phase system n-octanol and PBS.

### 4.2. Spectral Properties

Absorbance was measured with a Varian Cary 500 UV-VIS NIR Spectrophotometer (Agilent, Santa Clara, CA, USA) equipped with the software Cary 500 (EPROM Version 8.01, Agilent, Santa Clara, CA, USA) in the 200–1200 nm interval. Fluorescence emission was measured with a Varian Cary Eclipse Fluorescence-Spectrophotometer (Agilent, Santa Clara, CA, USA) equipped with the software Cary Eclipse (Version 1.1, Agilent, Santa Clara, CA, USA) with excitation at 710 nm in the 720–900 nm interval. PSMA-927 was diluted to 10 μM in PBS (pH 7.4) and measured in standard 1 cm quartz cuvettes.

### 4.3. Cell Binding and Internalization Properties

LNCaP cells (CRL-1740; ATCC; PSMA-positive) and PC-3 cells (CRL-1435; ATCC; PSMA-negative) were cultured in RPMI medium (Thermo Fisher Scientific, Waltham, MA, USA) supplemented with 10% fetal calf serum and 2 mmol/L L-glutamine (all from (Thermo Fisher Scientific, Waltham, MA, USA) ) at 37 °C in humidified air with 5% CO_2_. Cells were harvested using trypsin-ethylenediaminetetraacetic acid (trypsin-EDTA; 0.25% trypsin, 0.02% EDTA, Invitrogen, Waltham, MA, USA). Cell line authentication was regularly performed. Binding affinities and internalization profiles of the ^68^Ga-labeled compounds were determined in competitive cell binding assays and internalization experiments as described previously [17,23].

### 4.4. Confocal Microscopy

For confocal microscopy sample preparation, 10^5^ cells/well were seeded in poly-L-lysine (Sigma-Aldrich, Darmstadt, Germany) coated 4-well chambered coverslips (Nunc Lab-Tek II chambered coverglasses, Thermo Fisher Scientific, Waltham, MA, USA, live cell imaging) or on poly-L-lysine (Sigma-Aldrich, Darmstadt, Germany) coated #1 coverslips (15 mm diameter, fixed cell imaging) and kept for 48 h at 37 °C in humidified air with 5% CO_2_. Subsequently, cells were incubated with 50 nmol/L PSMA-927in RPMI for 5/15/30/45/60 min at 37 °C in humidified air with 5% CO_2_. After washing with PBS, cells were either subjected to live cell imaging in Gibco FluoroBrite DMEM (Thermo Fisher Scientific, Waltham, MA, USA) supplemented with 10% fetal calf serum and 4 mmol/L Gibco GlutaMAX^TM^ (Thermo Fisher Scientific, Waltham, MA, USA) or fixed with paraformaldehyde (2% PFA in PBS, both Thermo Fisher Scientific, Waltham, MA, USA) for 12 min and mounted in Mowiol (Sigma-Aldrich, Darmstadt, Germany).

All confocal data were acquired with a custom-built STED system similar to the one published by Gorlitz et al. [24]. A nanosecond pulsed 775 nm fiber laser (MPBC, usually employed as depletion laser for STED imaging) was used at 420 µW for excitation of PSMA-927, and a pulsed 405 nm diode laser (LDH-P-C400, PicoQuant, Berlin, Germany) was used at 60 µW for excitation of Hoechst and DAPI. Laser powers were measured in front of the microscope body. Dual color live (fixed) cell confocal images were acquired in line (frame) multiplexing mode with a pixel size of 100 nm, a dwell time of 10 μs, and a line accumulation of 3 (1).

Only images with a comparable number of cells per field of view were subjected to quantitative image analysis. The integrated fluorescence intensity was extracted with a custom-written ImageJ routine [25]. This script creates a mask for each image based on a fixed signal intensity threshold distinguishing between background (set to “not-a-number”) and signal (set to “1”). Multiplying this mask with the corresponding raw image generates an output image with which the integrated fluorescence intensity can be evaluated exclusively in the signal-carrying parts of the raw image without summing up background.

Background correction was solely done for the Hoechst/DAPI channel by subtracting at most 10% of the maximum fluorescence signal. Afterwards, linear deconvolution (Wiener filter) was applied to the same channel with a Lorentzian PSF (FWHM 200 nm) and only to the extent that data were smoothed and noise was reduced but resolution was not increased.

### 4.5. Cytotoxicity Study

Potential cytotoxicity of PSMA-927 was assessed by analyzing the frequency of LNCaP cell division and the length of the LNCaP cell cycle via holographic time-lapse imaging. Cells were seeded in lumox^®^ 24-well plates (Sarstedt, Nümbrecht, Germany) 48 h prior to the experiment and kept at 37 °C and 5% CO_2_. Imaging was performed with a HoloMonitor^®^ M4 cytometer (PHI AB, Lund, Schweden) for a total period of 48 h at 37 °C and 5% CO_2_ with 15 min between image captures. Cell proliferation was followed in absence and in presence of 100 nmol/L PSMA-927. Data analysis was performed with the AppSuite software including cell segmentation, tracking of cells, identification of dividing cells, confluency measurement, and cell counting.

### 4.6. Biodistribution and Preclinical Proof-of-Concept Study

Biodistribution and proof-of-concept imaging studies were performed in an experimental tumor xenograft model. A total of 5 × 10^6^ cells of LNCaP (in 50% Matrigel; Becton Dickinson, Franklin Lakes, NJ, USA) were subcutaneously inoculated into the flank of 7- to 8-week-old male BALB/c nu/nu mice (Charles River, Wilmington, MA, USA/Janvier Labs, Le Genest-Saint-Isle, France).

For biodistribution studies, 60 pmol of ^68^Ga-labeled PSMA-927 were injected into the tail vein (1–3 MBq, *n* = 3). The animals were sacrificed at 1 and at 2 h p.i., and organs of interest were dissected, blotted dry, and weighed. Finally, the radioactivity was measured using a gamma counter and calculated as % ID/g.

For the proof-of-concept imaging study, 0.5 nmol of the ^68^Ga-labeled compound in 0.9% NaCl (pH 7) were injected into the tail vein under anesthesia (2% isoflurane, *n* = 1). PET/MRI was performed with a 3T PET/MRI scanner (Bruker, Ettlingen, Germany). For MR imaging, a nontriggered localizer followed by a T1-weighted 3D scan was applied. For PET imaging, a dynamic scan for 60 min and a static scan (20 min) at 2 h p.i. was recorded. Image reconstruction was done with ParaVision software (dynamic scan: MLEM 0.5 mm algorithm, 12 iterations; static scan: OSEM 0.25 mm algorithm, 1 iteration), and data analysis was conducted in PMOD (version 3.7) with data converted to SUV images. After PET/MRI, the mice were sacrificed, and optical imaging of the subcutaneous tumor and organs of interest was performed with the Odyssey CLx system (LI-COR Biosciences, excitation wavelength 800 nm).

All animal experiments were approved by the regional authorities *Regierungspräsidium Karlsruhe* and *Regierungspräsidium Freiburg* and complied with the current laws of the Federal Republic of Germany.

### 4.7. Statistical Aspects

The experiments were performed at least in triplicate except for the imaging proof-of-concept study (*n* = 1). Quantitative data are expressed as mean ± standard deviation (SD) with the number of replicates n given in the respective figure or table captions. Bar plots depict the mean ± SD of the measurements for replicate experiments, while box plots indicate the interquartile range (box), the outer-most data points falling within 1.5× interquartile range (whiskers), the median (center line), and the mean (triangle) of the measurements for replicate experiments. If applicable, means were compared using Student’s *t* test (GraphPad Prism Version 8, GraphPad Software, Inc., San Diego, CA, USA). *p*-values < 0.05 were considered statistically significant.

## 5. Conclusions

This study presents the development and preclinical characterization of a novel class of PSMA-targeting hybrid molecules based on the theranostic radiopharmaceutical PSMA-617 for pre- and intraoperative detection of prostate cancer. By strategically analyzing different molecule compositions, we addressed the highly challenging introduction of a bulky dye to a low-molecular-weight peptidomimetic and uncovered structure–activity relationships with influence on the internalization profile of the hybrid molecules. With the final lead candidate PSMA-927 featuring properties comparable to those of PSMA-617 in a preclinical proof-of-concept setting, this novel class of PSMA-targeting hybrid molecules warrants subsequent investigations and a potential clinical translation. The new insights uncovered by our study provide a valuable contribution to further advance the field of targeted peptidomimetic hybrid molecule development.

## Figures and Tables

**Table 1:** Cell binding and internalization data of all candidates of the hybrid molecule library *.

Compound	Specifically Cell Surface Bound [%AR/10^5^ Cells] ^†^	Specifically Internalized [%AR/10^5^ Cells] ^†^	$IC_{50}[\mathrm{nmol}/L]{\;}^{‡}\;\mathrm{Free}\;\mathrm{Ligands}$	$K_{i}[\mathrm{nmol}/L]{\;}^{‡}\;\mathrm{Free}\;\mathrm{Ligands}$
PSMA-921	7.06 ± 3.32	15.82 ± 6.18	4.83 ± 0.93	4.03 ± 0.95
PSMA-922	6.33 ± 1.53	13.74 ± 5.75	6.93 ± 1.29	5.78 ± 1.32
PSMA-923	9.82 ± 0.67	21.55 ± 9.24	7.14 ± 1.95	5.95 ± 1.99
PSMA-924	1.11 ± 0.81	1.11 ± 0.94	4.64 ± 1.23	3.87 ± 1.76
PSMA-925	2.67 ± 0.95	3.03 ± 2.17	8.12 ± 3.59	6.77 ± 3.66
PSMA-926	10.12 ± 4.85	11.86 ± 5.31	8.59 ± 2.57	7.16 ± 2.63
PSMA-927	25.51 ± 9.73	27.64 ± 12.80	15.87 ± 5.46	13.22 ± 5.25

* Data are expressed as mean ± SD (*n* = 3), ^† 68^Ga-labeled compounds. Specific cell uptake was determined by blockage using 500 µmol/L 2-PMPA. Values are expressed as % of applied radioactivity (AR) bound to 10^5^ cells. ^‡^ Radioligand: ^68^Ga-PSMA-10 ($K_{d}$: 3.8 ± 1.8 nmol/L [17], $c_{radioligand}$: 0.75 nmol/L), $K_{i}=IC_{50}/\left(1+c_{radioligand}/K_{d}\right)$.

## Data Availability

Data is contained within the article and Supplementary Material.

## Supplementary Materials

The following are available online at https://www.mdpi.com/article/10.3390/ph15030267/s1, Supplemental Scheme S1: Synthesis of Glu-urea-Lys-2-Nal-Chx-Lys-Ei-DOTA (i = 0–2) according to standard Fmoc solid phase protocols, Supplemental Scheme S2: Synthesis of Glu-urea-Lys-2-Nal-Chx-Lys(DOTA)-NH_2_ according to standard Fmoc solid phase protocols, Supplemental Scheme S3: Synthesis of Glu-urea-Lys-2-Nal-Chx-Lys(DOTA)-Ei (i = 1–2) according to standard Fmoc solid phase protocols, Supplemental Figure S1: Radio-Analytical data of ^68^Ga-labeledPSMA-927, Supplemental Table S1: Analytical data of all candidates of the hybrid molecule library, Supplemental Table S2: Organ distribution of 0.06 nmol ^68^Ga-labeled PSMA-927 in LNCaP-tumor bearing BALB/c nu/nu mice 1 h p.i., Supplemental Table S3: Organ distribution of 0.06 nmol ^68^Ga-labeled PSMA-927in LNCaP-tumor bearing BALB/c nu/nu mice 2 h p.i., Movie S1: LNCaP control, Movie S2: LNCaP 100nM compound.
