# Supplementary material for: A New Class of PSMA-617-Based Hybrid Molecules for Preoperative Imaging and Intraoperative Fluorescence Navigation of Prostate Cancer
Source: Pharmaceuticals (Basel). 2022 Feb 22;15(3):267. doi: 10.3390/ph15030267 (PMC8954540; doi:10.3390/ph15030267)
Supplement: Supplementary file 1 [file pharmaceuticals-15-00267-s001.zip › pharmaceuticals-1544305-supplementary.pdf]

Supplemental Information

A New Class of PSMA-617-based Hybrid Molecules for Preoperative Imaging and Intraoperative Fluorescence Navigation of Prostate Cancer

Ann-Christin Eder[1,2,3]*, Jessica Matthias[4], Martin Schäfer[1], Jana Schmidt[1], Nils Steinacker[2,3], Ulrike Bauder-Wüst[1], Lisa-Charlotte Domogalla[2,3], Mareike Roscher[1], Uwe Haberkorn[5,6], Matthias Eder[2,3,†], Klaus Kopka[1,7,8,9,†]

Compound Synthesis and Characterization 2

Supplemental Schemes 4

Supplemental Scheme S1. Synthesis of Glu-urea-Lys-2-Nal-Chx-Lys-E_i-DOTA (i=0-2) according to standard Fmoc solid phase protocols. 4
Supplemental Scheme S2. Synthesis of Glu-urea-Lys-2-Nal-Chx-Lys(DOTA)-NH_2 according to standard Fmoc solid phase protocols. 5
Supplemental Scheme S3. Synthesis of Glu-urea-Lys-2-Nal-Chx-Lys(DOTA)-E_i (i=1-2) according to standard Fmoc solid phase protocols. 6

Supplemental Figures 7

Supplemental Figure S1. Radio-Analytical data of ^{68}Ga-labeled Glu-urea-Lys-2-Nal-Chx-Lys(IRDye800CW)-DOTA. 7

Supplemental Tables 8

Supplemental Table S1. Analytical data of all candidates of the hybrid molecule library. 8
Supplemental Table S2. Organ distribution of 0.06 nmol ^{68}Ga-labeled Glu-urea-Lys-2-Nal-Chx-Lys(IRDye800CW)-DOTA in LNCaP-tumor bearing BALB/c nu/nu mice 1 h p.i.* 9
Supplemental Table S3. Organ distribution of 0.06 nmol ^{68}Ga-labeled Glu-urea-Lys-2-Nal-Chx-Lys(IRDye800CW)-DOTA in LNCaP-tumor bearing BALB/c nu/nu mice 2 h p.i.* 9

References 10

Compound Synthesis and Analytical Characterization

All commercially available chemicals were of analytical grade and used without further purification. Compounds were purified with the preparative HPLC system LaPrep P110 (VWR) equipped with a variable UV detector (P314, VWR). For preparative purification, a NUCLEODUR® Sphinx RP column (VP250/21, 5 µm 250 x 21 mm; Macherey-Nagel) was used with a 20 min linear gradient from 10% B to 100% B and a flow rate of 20 mL/min (A: 0.1% aqueous TFA, B: 0.1% TFA in CH_3CN). Semipreparative and analytical HPLC runs were performed with the system Agilent 1100 series (Agilent Technologies) equipped with Chromolith RP-18e, 100×10mm/Chromolith RP-18e, 100×4.6 mm (Merck) columns. UV absorbance was measured at 214 nm and 254 nm. Semipreparative HPLC purifications of the final products were conducted with a linear gradient (100% A: 0.1% aqueous TFA to 100% B: 0.1% TFA in CH_3CN) in 10 min at 5 mL/min. For analytical HPLC runs, a linear gradient (100% A: 0.1% aqueous TFA to 100% B: 0.1% TFA in CH_3CN) in 10 min at 2 mL/min was employed. For all final products, the chemical purity was greater than 98% as determined by HPLC. Mass spectrometry was performed with a MALDI-MS (Daltonics Microflex, Bruker Daltonics) and 2,5-dihydroxybenzoic acid as matrix.

Synthesis of Glu-urea-Lys-2-Nal-Chx-Lys(FITC)-E_i-DOTA, Glu-urea-Lys-2-Nal-Chx-Lys(DOTA)-E_i-FITC (i=0-2) and Glu-urea-Lys-2-Nal-Chx-Lys(IRDye800CW)-DOTA

In a first step, the pharmacophore Glu-urea-Lys was synthesized as described previously (*1*). The formation of the isocyanate of the glutamyl moiety was performed using triphosgene. Afterwards, a resin-immobilized (2-chloro-tritylresin), ε-allyloxycarbonyl protected lysine was added and stirred for 16 h at room temperature (RT) with gentle agitation. Finally, the resin was filtered off and the allyloxycarbonyl-protecting group was removed by reacting twice with $Pd(PPh_3)_4$ (0.3 eq.) and morpholine (15 eq.) in dichloromethane under ambient conditions (1 h, RT, light protected).

The following synthesis steps for linker introduction were performed using standard Fmoc solid phase protocols. In the first step and for all compounds, Fmoc-2-Nal-OH and N-Fmoc-

tranexamic acid (4 eq. each) were coupled in DMF with HBTU and DIPEA (4 eq. each), respectively. Afterwards the resin was split.

For synthesis of Glu-urea-Lys-2-Nal-Chx-Lys(Boc)-E_i-DOTA (i=0-2), Fmoc-Lys(Boc)-OH (**5**) was directly reacted with HBTU (4 eq. each) in DMF, and for synthesis of the linker unit E_i (i=0) (**6**), tris(t-Bu)DOTA (tris(tBu)-ester of 1,4,7,10-tetraazacyclododecan-1,4,7,10-tetraacetic acid) was directly reacted with DIPEA (4 eq. each) in DMF (Supplemental Scheme 1).

For introduction of the linker unit E_i (i=1-2) (**7**)-(**8**), Fmoc-Glu(Ot-Bu)-OH (4 eq.) was coupled once or twice with HBTU (4 eq.) and DIPEA (4 eq.) in DMF followed by coupling with tris(t-Bu)DOTA.

To obtain Glu-urea-Lys(2-Nal-Chx-Lys(DOTA)-Boc) (**9**), Boc-Lys(Alloc)-OH (4 eq.) was reacted with HBTU (4 eq.) and DIPEA (4 eq.) in DMF, and subsequently the Alloc protecting group was removed using $Pd(PPh_3)_4$ (0.3 eq.) and morpholine (15 eq.) in dichloromethane. Afterwards, tris(t-Bu)DOTA (4 eq.) was coupled with HBTU (4 eq.) and DIPEA (4 eq.) in DMF (**10**) (Supplemental Scheme 2).

For the synthesis of Glu-urea-Lys(2-Nal-Chx-Lys(DOTA)-E_i-Boc) (i=1-2), Fmoc-Lys(Alloc)-OH, HBTU and DIPEA (4 eq. each) were mixed in DMF (**11**). For i=1, Boc-Glu(Ot-Bu)-OH and after Alloc protecting group removal tris(t-Bu)DOTA were reacted with HBTU and DIPEA (all 4 eq.) in DMF (**12**). For i=2, Fmoc-Glu(Ot-Bu)-OH, Boc-Glu(Ot-Bu)-OH (**14**) and after Alloc protecting group removal tris(t-Bu)DOTA were reacted with HBTU and DIPEA (all 4 eq.) in DMF (**13** and **15**) (Supplemental Scheme 3).

The products were cleaved from the resin for 3 h at RT using TFA/TIPS/H2O (95/2.5/2.5, v/v/v). Fluorescein isothiocyanate (FITC; 1 eq.) was dissolved in 50 µL DMSO and conjugated with the previously synthesized precursors Glu-urea-Lys-2-Nal-Chx-Lys-E_i-DOTA (i=0-2) and Glu-urea-Lys(2-Nal-Chx-Lys(DOTA)-E_i-NH_2) (i=0-2) (3 eq. each) in DMF (300 µL) and DIPEA (15 µL) for 24 h at RT. IRDye800CW-NHS ester (LI-COR Biosciences; 1 eq.) was conjugated to Glu-urea-Lys-2-Nal-Chx-Lys-DOTA in PBS-buffer (pH 8.5) for 24h at RT. The final products were isolated via semipreparative HPLC and identified with mass spectrometry.

Supplemental Schemes

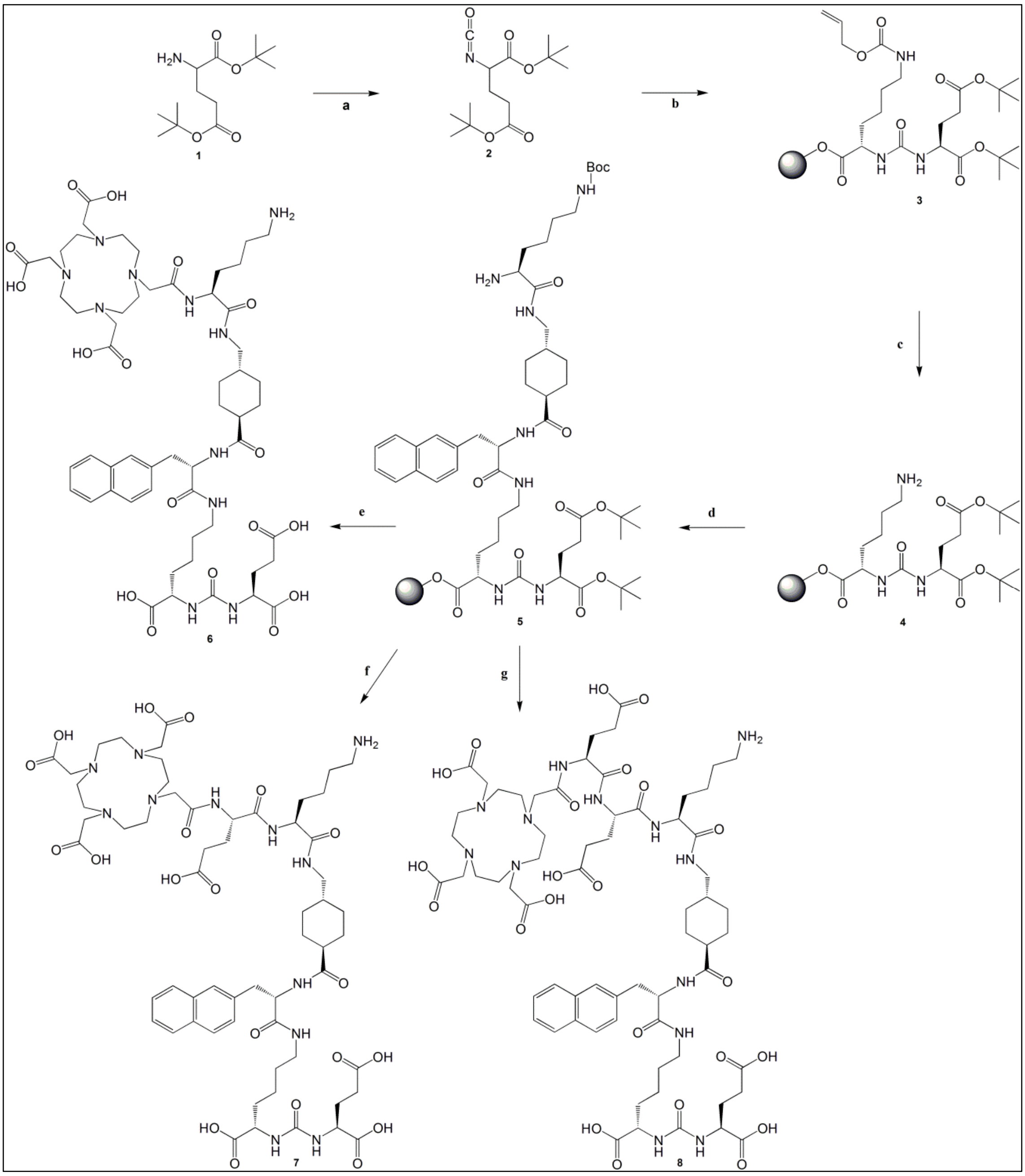

Supplemental Scheme S1. Synthesis of Glu-urea-Lys-2-Nal-Chx-Lys-E_i-DOTA (i=0-2) according to standard Fmoc solid phase protocols. (**a**) Triphosgene in DCM und DIPEA, 4 °C, 4 h, (**b**) H-Lys(Alloc)-2-CT-resin, DCM, 16 h, RT, (**c**) 0.3 eq. TPP palladium(0), 15 eq. morpholin in DCM, RT, 1 h, light protected, (**d**) Fmoc-2-Nal-OH, N-Fmoc-tranexamic acid, Fmoc-Lys(Boc)-OH (4 eq. each), DMF, HBTU, DIPEA, 50 % piperidine in DMF, (**e**) tris(*t*-Bu)DOTA (4 eq.), DMF, DIPEA, (**f**) Fmoc-Glu(*t*-Bu)-OH, 50 % piperidine in DMF, tris(*t*-Bu)DOTA (4 eq. each), DMF, HBTU, DIPEA, TFA/TIPS/H_2O, (**g**) Fmoc-Glu(*t*-Bu)-OH, Fmoc-Glu(*t*-Bu)-OH, 50 % piperidine in DMF, tris(*t*-Bu)DOTA (4 eq.), DMF, HBTU, DIPEA, TFA/TIPS/H_2O.

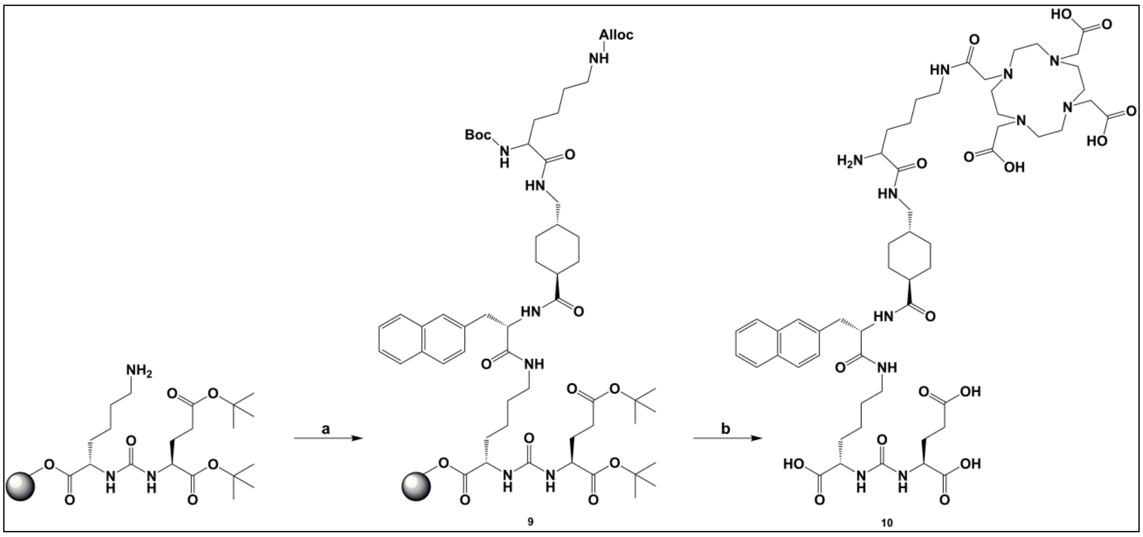

Supplemental Scheme S2. Synthesis of Glu-urea-Lys-2-Nal-Chx-Lys(DOTA)-NH_2 according to standard Fmoc solid phase protocols. (**a**) Fmoc-2-Nal-OH, N-Fmoc-tranexamic acid, Boc-Lys(Alloc)-OH (4 eq. each), DMF, HBTU, DIPEA, 50 % piperidine in DMF, (**b**) 0.3 eq. TPP palladium(0), 15 eq. morpholine in DCM, RT, 1 h, light protected, tris(*t*-Bu)DOTA (4 eq.), DMF, DIPEA, HBTU, TFA/TIPS/H_2O.

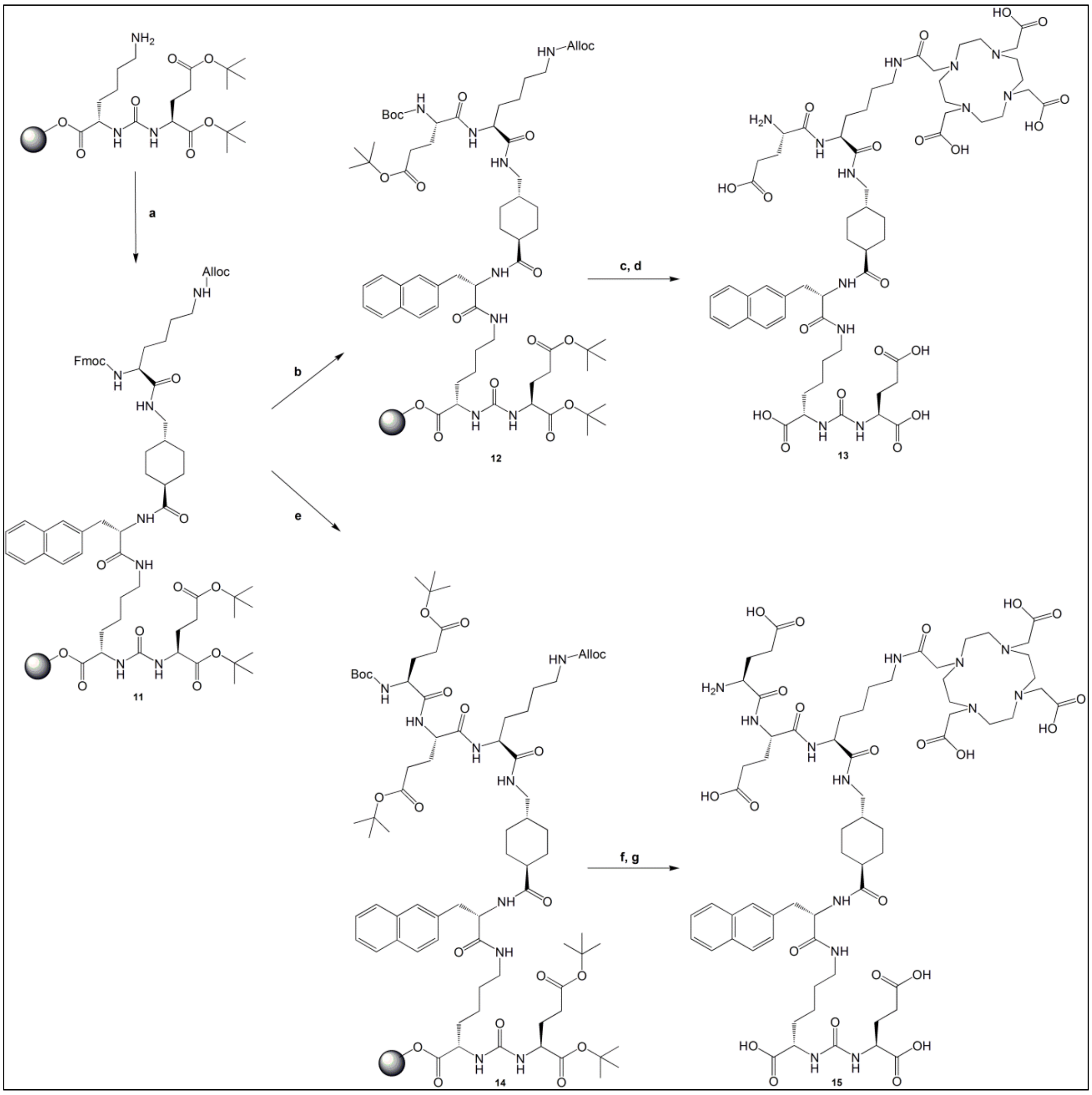

Supplemental Scheme S3. **Synthesis of Glu-urea-Lys-2-Nal-Chx-Lys(DOTA)-E_i (i=1-2) according to standard Fmoc solid phase protocols.** (**a**) Fmoc-2-Nal-OH, N-Fmoc-tranexamic acid, Fmoc-Lys(Alloc)-OH (4 eq. each), DMF, HBTU, DIPEA, 50 % piperidine in DMF, (**b**) 50 % piperidine in DMF, Boc-Glu(*t*-Bu)-OH (4 eq.), DMF, HBTU, DIPEA, (**c**) 0.3 eq. TPP palladium(0), 15 eq. morpholine in DCM, RT, 1 h, light protected, (**d**) tris(*t*-Bu)DOTA (4 eq.), DMF, DIPEA, HBTU, TFA/TIPS/H_2O, (**e**) Fmoc-Glu(*t*-Bu)-OH, 50 % piperidine in DMF, Boc-Glu(*t*-Bu)-OH (4 eq. each), DMF, HBTU, DIPEA, (**f**) 0,3 eq. TPP palladium(0), 15 eq. Morpholin in DCM, RT, 1 h, light protected, (**g**) tris(*t*-Bu)DOTA (4 eq.), DMF, DIPEA, HBTU, TFA/TIPS/H_2O.

Supplemental Figures

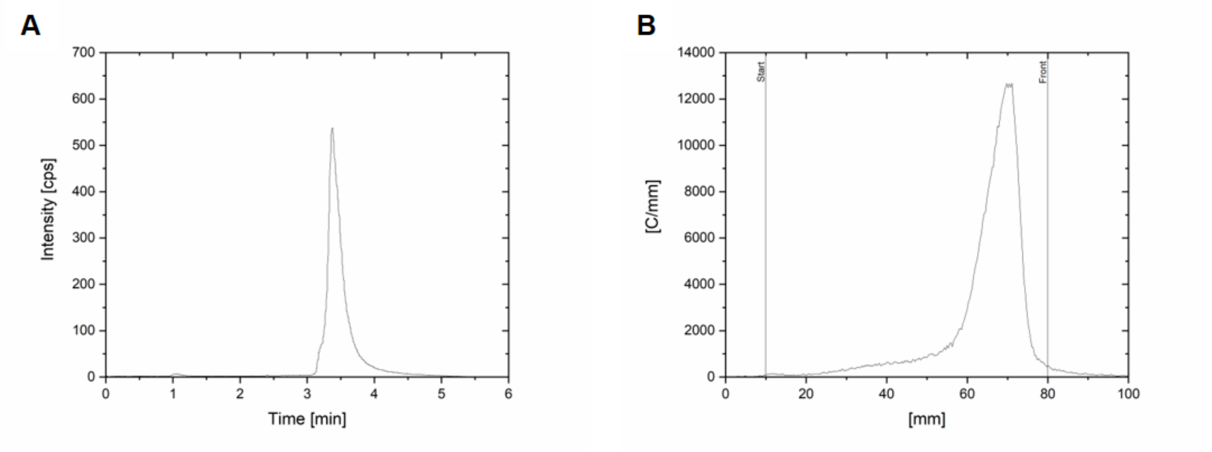

Supplemental Figure S1. Radio-Analytical data of ^{68}Ga-labeled Glu-urea-Lys-2-Nal-Chx-Lys(IRDye800CW)-DOTA. (**A**) Radio-analytical reversed-phase HPLC of the ^{68}Ga-labled compound was performed using a Chromolith RP-18e 100x4.6 mm column with a linear gradient (10 % A (0.1% TFA in H_2O) to 90% B (0.1% TFA in CH_3CN)) in 6 min at 2 mL/min. (**B**) RP-TLC (60 RP-18 F_{254S}) of the ^{68}Ga-labled compound was performed with 0.5 M ammonium acetate/methanol (1:1) as mobile phase. Original data was plotted using OriginPro 2020 software.

Supplemental Tables

Supplemental Table S1. Analytical data of all candidates of the hybrid molecule library.

Compound	Sum formula	m/z<br><br>exact mass (calc.)	m/z*	Chemical yield [%]†	Lipophilicity $\log D_{pH\ 7.4}$ *n-octanol/PBS*
Glu-urea-Lys-2-Nal-Chx-Lys(**Fluorescein**)-DOTA	$C_{76}H_{94}N_{12}O_{22}S$	1558.6	1560.8	53	-2.36 ± 0.03
Glu-urea-Lys-2-Nal-Chx-Lys(**Fluorescein**)-E_1-DOTA	$C_{81}H_{101}N_{13}O_{25}S$	1687.7	1689.6	38	-2.07 ± 0.06
Glu-urea-Lys-2-Nal-Chx-Lys(**Fluorescein**)-E_2-DOTA	$C_{86}H_{108}N_{14}O_{28}S$	1816.7	1819.1	31	-2.16 ± 0.04
Glu-urea-Lys-2-Nal-Chx-Lys(DOTA)-**Fluorescein**	$C_{76}H_{94}N_{12}O_{22}S$	1559.7	1559.9	31	-2.38 ± 0.07
Glu-urea-Lys-2-Nal-Chx-Lys(DOTA)-E_1- **Fluorescein**	$C_{81}H_{101}N_{13}O_{25}S$	1688.8	1689.7	39	-2.05 ± 0.02
Glu-urea-Lys-2-Nal-Chx-Lys(DOTA)-E_2-**Fluorescein**	$C_{86}H_{108}N_{14}O_{28}S$	1817.9	1818.7	35	-2.13 ± 0.03
Glu-urea-Lys-2-Nal-Chx-Lys(**IRDye800CW**)-DOTA	$C_{101}H_{136}N_{13}O_{31}S_4^+$	2154.8	2157.8	46	-2.96 ± 0.06

* Mass spectrometry of non-labeled ligand detected as $[M+H]^+$, † Chemical yields refer to the fluorescent dye conjugation, ‡ Lipophilicity of ^{68}Ga-labeled ligand.

Supplemental Table S2. Organ distribution of 0.06 nmol ^{68}Ga-labeled Glu-urea-Lys-2-Nal-Chx-Lys(IRDye800CW)-DOTA in LNCaP-tumor bearing BALB/c nu/nu mice 1 h p.i.*.

	^{68}Ga-Glu-urea-Lys-2-Nal-Chx-Lys(IRDye800CW)-DOTA [%ID/g]	^{68}Ga-Glu-urea-Lys-2-Nal-Chx-Lys(IRDye800CW)-DOTA Tumor-to-Organ ratio
Blood	1.14 ± 0.14	3.69 ± 0.60
Heart	0.51 ± 0.10	8.57 ± 2.07
Lung	1.47 ± 0.14	2.84 ± 0.37
Spleen	2.82 ± 1.19	1.76 ± 0.71
Liver	0.95 ± 0.15	4.51 ± 0.84
Kidney	65.64 ± 6.60	0.06 ± 0.01
Muscle	0.34 ± 0.03	12.23 ± 1.39
Intestine	0.43 ± 0.12	10.37 ± 2.47
Brain	0.07 ± 0.01	57.84 ± 9.25
Tumor	4.13 ± 0.15	-

* Data are expressed as mean % ID/g tissue ± SD (n=3).

Supplemental Table S3. Organ distribution of 0.06 nmol ^{68}Ga-labeled Glu-urea-Lys-2-Nal-Chx-Lys(IRDye800CW)-DOTA in LNCaP-tumor bearing BALB/c nu/nu mice 2 h p.i.*.

	^{68}Ga-Glu-urea-Lys-2-Nal-Chx-Lys(IRDye800CW)-DOTA [%ID/g]	^{68}Ga-Glu-urea-Lys-2-Nal-Chx-Lys(IRDye800CW)-DOTA Tumor-to-Organ ratio
Blood	0.82 ± 0.23	7.58 ± 1.65
Heart	0.43 ± 0.14	14.79 ± 4.40
Lung	1.11 ± 0.42	6.11 ± 2.36
Spleen	2.08 ± 0.90	3.60 ± 1.86
Liver	0.87 ± 0.34	7.80 ± 3.09
Kidney	57.47 ± 23.83	0.13 ± 0.06
Muscle	0.66 ± 0.30	11.55 ± 5.94
Intestine	0.59 ± 0.26	11.92 ± 5.17
Brain	0.24 ± 0.11	29.37 ± 13.17
Tumor	5.85 ± 0.91	-

* Data are expressed as mean % ID/g tissue ± SD (n=3).

References

1. Schafer M, Bauder-Wust U, Leotta K, et al. A dimerized urea-based inhibitor of the prostate-specific membrane antigen for 68Ga-PET imaging of prostate cancer. *EJNMMI Res.* 2012;2:23.
